# Detection of Math6-Expressing Cell Types in Murine Placenta

**DOI:** 10.3390/biology12091252

**Published:** 2023-09-19

**Authors:** Maren Brendel, Marion Scharf, Urs Kindler, Satya Srirama Karthik Divvela, Beate Brand-Saberi

**Affiliations:** Department of Anatomy and Molecular Embryology, Institute of Anatomy, Medical Faculty, Ruhr University Bochum, 44801 Bochum, Germany; maren.brendel@ruhr-uni-bochum.de (M.B.); marion.scharf@ruhr-uni-bochum.de (M.S.); urs.kindler@ruhr-uni-bochum.de (U.K.); satya.divvela@ruhr-uni-bochum.de (S.S.K.D.)

**Keywords:** Math6, mouse placenta, Flag-tag, immunohistochemistry, fluorescence-activated cell sorting, endothelial cells, uNK, TGCs, macrophages

## Abstract

**Simple Summary:**

The protein, mouse atonal homolog 6 (Math6), is required for correct placental development in mice and causes loss of pregnancy, leading to reproductive insufficiency in females, when it is missing. It is currently unclear which cell types of the placenta predominantly express this protein. With the help of transgenic Math6 Flag-tag mice, we established two methods to characterize the Math6-positive cells: immunohistochemical staining and cell sorting. Using these methods, we discovered that Math6 protein is expressed in multiple layers of the murine placenta in diverse cell types: decidua cells, uterine natural killer cells, macrophages, trophoblast giant cells, and endothelial cells.

**Abstract:**

The transcription factor Math6, mouse atonal homolog 6, belongs to the family of highly conserved basic helix–loop–helix transcription factors. It plays an important role in embryonic development and shows a wide expression pattern in murine tissues. The placenta, as a life-sustaining transient organ for the fetus, also depends on the expression of Math6. The adverse effects of deleting Math6 in mice, leading to deficient placental development and pregnancy loss, have already been demonstrated by us. Until now, detailed investigations regarding the specific mechanisms underlying the improper placental development in these murine mutants have failed, as the Math6 expression could not be confined to a specific cell type due to the lack of a highly specific Math6 antibody. To circumvent this problem, we used transgenic mice, where Math6 is marked with a Flag sequence that functions as a specific epitope. Tissues from these transgenic mice were used to establish immunohistochemical staining and fluorescence-activated cell sorting (FACS). The establishment of these methods yielded initial findings pertaining to the identification of Math6-expressing cell types and their localization. Our results reveal that Math6 shows a wide expression pattern in both maternal and fetal components of the murine placenta. It shows expression in various cell types, but predominantly in trophoblast giant cells, endothelial cells and macrophages. The largest subpopulation that we detected in the group of Math6-positive cells were identified as DBA+ uterine natural killer cells. These findings reveal information and a chance for further investigation on the involvement of Math6 in placental development and the molecular pathomechanisms of spontaneous abortion.

## 1. Introduction

The mouse is a suitable and well-described model organism for studying pathologies in placental development, which allows us to work with genetically modified mice. The collection of placental tissue is possible at different developmental stages and is associated with fewer ethical concerns than human tissue experiments.

The uterine side of placental development, called the mesometrial side, is located medially where the blood vessels enter and run parallel to the uterus horns. During pregnancy, multiple placentas develop in each uterine horn, along with each fetus [1].

After fertilization in the Fallopian tube, the zygote undergoes rapid cell division and becomes the blastocyst four days later. In this stage of development and after arrival in the uterus, the blastocyst implants into the antimesometrial side of the uterine wall [2]. The luminal epithelium and its associated basement membrane undergo apoptosis in order to allow the trophectoderm-derived trophoblast cells to extend into the stroma [3]. Decidualization is triggered by the interaction between the implanted embryo and luminal epithelium [4]. During decidualization, that starts near the implanted embryo and then continues to spread, the endometrial stromal cells proliferate into decidual cells to form the maternal component of the placenta. For this purpose, they increase in cell size and accumulate glycogen and lipids that provide a source of nutrients for the fetus [5].

Another important step, ensuring the embryo’s nutrition and oxygen supply and meet its high metabolic demands, is the vascular remodeling that is regulated by the fetal trophoblast cells. Through secretion of cytokines and proapoptotic factors, they partly replace the smooth muscle cells of the spiral arteries with the aim of enlarging the diameter and lowering the vascular resistance of the arteries [6]. An important subtype are the trophoblast giant cells (TGCs), which form the barrier between maternal and fetal tissues of the placenta [7]. Uterine natural killer cells (uNK) are likewise involved in associated processes, such as vascular remodeling and angiogenesis. These are specialized immune cells that form the majority of leukocytes in the murine placenta during pregnancy, secreting VEGF and INF-γ [8]. A subtype of these uNK cells can be detected by using a specific marker called *Dolichos biflorus* agglutinin (DBA) [9]. During pregnancy, the immune system in general is modulated to prevent the immunological rejection of the embryo. Immune cells, like uNK and macrophages, detected by the surface marker, F4/80 [10], are therefore more abundant in the placenta [11]. They form a special tissue called the mesometrial lymphoid aggregate of pregnancy (MLAp). The MLAp is a transient tissue rich in immune cells, which develops unencapsulated within the uterine wall.

In summary, precise regulation, including the involvement of transcription factors like Math6, is necessary for the multitude of intricate and interacting processes during placental development. Basic helix–loop–helix (bHLH) transcription factors can impact gene expression by repressing or promoting it through DNA-binding, and therefore they play a key role in a variety of developmental processes [12]. Math6 has been identified in not only the murine placenta but in several organs and organisms, including humans, as the bHLH domain of the gene is highly conserved across many species [13].

In mice, Math6 appears to be essential for the reproductive success of the species. While Math6 homozygous knockout mice can survive, they exhibit noticeable phenotypic alterations. In comparison to heterozygote animals, female Math6-knockout mice are unable to conceive viable offspring, independent of the paternal phenotype. Because prenatal miscarriages are mainly caused by the maternal phenotype, the expression of Math6 in the maternal decidua of the placenta is expected. During pregnancy, they show deficient placenta development that is most prominently characterized by a misshapen decidua basalis, even though the area remains the same size and decidua cells are not altered in number. In contrast, some cell types, like uNK, decrease in number, while others, like TGCs, increase. Vascularization is equally deficient, which leads to critical complications including uterine hemorrhaging on gestational day 13.5. Math6 influences the RNA expression of many markers, for example, vascularization marker *CD31*, which is significantly reduced in knockout mice. In another study, Math6 was also identified and investigated as an endothelial-selective and shear-stress-responsive transcription factor [14]. Overall, this leads to improper delivery and death of the offspring, which renders female homozygous knockout mice unable to continue pregnancy until term. This highlights the critical role of Math6 in ensuring accurate and complete placental development and maintenance [15].

Expression of Math6 in the placenta has previously been demonstrated using two methods. PCR has been used to demonstrate the RNA expression levels and showed that *Math6* is highly expressed on day E8.5 and then decreases later in pregnancy. In situ hybridization first revealed information on the *Math6* expression pattern [15]. Considering the high relevance of Math6 throughout placental development, we aimed to establish methods to identity and characterize Math6-expressing cell types. To overcome the lack of highly specific antibodies against Math6, we utilized transgenic Flag-mice. These mice, generated and established in collaboration with the School of Life Science of Tongji University, were already used to demonstrate the importance of Math6 for cellular reprogramming and early differentiation [16]. In detail, it could be shown that the knockout of Math6 in fibroblasts leads to incomplete mesenchymal-to-epithelial transitions during reprogramming, and that naive pluripotent iPSCs fail to maintain their pluripotency when lacking Math6.

In this study, Math6 Flag-tag transgenic mice were used for the establishment of two method protocols to detect Math6 in histological sections and in single-cell suspensions of placental tissues at two developmental stages. A newly introduced immunohistochemical staining protocol enabled the characterization of Math6-expressing cell types as well as their localization. FACS analysis additionally confirmed the majority of Math6+ cells.

The aim of the study was to characterize the cell types that predominantly express Math6, thereby providing the possibility for further investigations of Math6 in the murine placenta. This information could us help understand the molecular pathomechanisms and possible misregulated crosstalk between cells in the placentas from Math6-knockout mice.

## 2. Materials and Methods

### 2.1. Flag-Mice

To overcome the lack of a highly specific antibody against Math6, transgenic mice with a Flag-tag were used. The knock-in of a 3× FLAG sequence (DYKDDDDK) at the C-terminus of the Math6 protein was achieved through CRISPR-CAS9 technology in C57BL/6 mice, as described by Divvela et al. [16]. To verify the integration of the sequence in the gene locus, PCR genotyping and DNA sequencing were performed [16]. These homozygous Flag-mice exhibited phenotypically wildtype characteristics with no detectable alterations and are capable of normal reproduction.

### 2.2. Mice Mating and Tissue Harvesting

The mice were kept in the medical faculty of the Ruhr-University Bochum in ventilated rooms with a room temperature of 21 ± 1 °C and a humidity of 55 ± 5 %. The mice were mostly caged together with 3 to 5 animals and fed rat/mouse housing food (V1534-000) from sniff (Soest, Germany). Within our mouse facility, animal experiments were carried out in compliance with the regulations of the Tierschutzgesetz in Germany and other animal protection regulations that are effective within the European Union.

Female homozygous Flag-mice from 18 to 25 weeks old were placed in a cage together with an age-matched homozygous male Flag-mouse overnight and separated the next morning. Observation of the vaginal plug in the morning was assumed as day E0.5 of fetal and placental development. Mice were killed via cervical dislocation at E10.5 or E13.5, and the uterus was obtained with all implantation sites. After being rinsed in 4 °C phosphate-buffered saline (PBS), the placentas and the associated embryo were separated from the other tissues. The German Animal Welfare Act was complied with under all circumstances.

### 2.3. Histology–Preparation of Sections and HE-Staining

Placentas were fixed in 45 °C warm 4% paraformaldehyde (PFA) in PBS immediately after collection. On E10.5, the entire implantation site, including placenta and embryo, was fixed. However, on E13.5, the placenta was large enough to remove the embryo without morphological damage. After 30 min, the PFA was cooled to 4 °C and stored for a maximum of 24 h. Once rinsed with PBS, the samples were slowly dehydrated with ethanol over the course of two days and finally embedded in paraffin. Sections of 5 μm thickness were produced by making medio-sagittal sections through the umbilical cord. Following dewaxing with RotiHistol (Carl Roth, Karlsruhe, Germany), the sections were rehydrated. Afterwards, they were subjected to hematoxylin–eosin (HE) staining via a 15 min treatment with hematoxylin followed by eosin for 2 min (Carl Roth, Karlsruhe, Germany). HE-stained sections were scanned with a virtual slide microscope VS120 (Olympus, Tokyo, Japan).

### 2.4. Immunohistochemical Analysis

For the purpose of antigen unmasking, the sections were deparaffinized and rehydrated before being immersed in 19 mM citrate buffer. This was brought to boiling temperature in the microwave followed by a constant heat supply over 15 min. Once cooled down to room temperature, the sections were rinsed with PBS and then treated with 0.1 M glycine followed by 1% Triton for 10 minutes. Blocking was performed using 10% fetal goat serum for one hour. The primary anti-Flag antibody (F1804, Sigma-Aldrich, Taufkirchen, Germany) was diluted 1:250 in the blocking solution and left to incubate on the slides overnight at 4 °C. After multiple washing steps with PBS, the slides were incubated with the secondary fluorescent antibodies Alexa Fluor goat anti-mouse 568 or Alexa Fluor goat anti-mouse 488 (Thermo Fisher, Waltham, MA, USA) for one hour. The sections were covered with cover slips and 4′,6-diamidino-2-phenylindol (DAPI) (Carl Roth, Karlsruhe, Germany) for nuclear staining. All immunohistochemical sections were scanned with the LSM 800 or Axio Scan.Z1 (Carl Zeiss, Oberkochen, Germany).

### 2.5. Fluorescence-Activated Cell Sorting (FACS)

Placentas collected on day E10.5 of the pregnancy were placed in PBS on ice immediately. Most of the myometrium and each embryo were then separated from the placenta individually, and each placenta tissue was placed in 500 μL of accutase (Thermo Fisher Scientific, Braunschweig, Germany). After 2 min of mincing the tissue with scissors, 500 µL of accutase was added on top. All samples were then incubated at 37 °C for 30 min. Afterwards, two placentas were filtered through a 40 μm cell strainer, which was then rinsed with PBS. Following centrifugation, the supernatant was removed, and the pellet was dissolved in PBS. The cells were treated as described in Table 1, with each step performed on ice. The FACS-buffer was prepared with a final concentration of 2% BSA, 2 mM EDTA and 1× PBS. FACS-buffer with 0.1% Triton X-100 (Sigma-Aldrich, Taufkirchen, Germany) was used as a permeabilization solution and to dilute the antibody against the intracellular antigen. All FACS antibodies were purchased from Biolegend (San Diego, CA, USA) and used in their recommended concentrations. Anti-CD31 antibody was used to detect endothelial cells, and anti-F4/80 antibody was used to identify macrophages. DBA lectin was purchased from vector laboratories (Newark, CA, USA) and used for the detection of uNK.

After the final staining, the cells were rinsed multiple times with FACS-buffer and subsequently preserved in it at 4 °C until analysis the next day. All samples were analyzed using a Beckman Coulter Cytoflex LX (Krefeld, Germany).

### 2.6. Data Analysis and Processing

FACS data were captured using Summit 6.3.1. and then processed with Kaluza Analysis 2.1. (Beckman Coulter, Inc., Krefeld, Germany). Isotype-stained and negative controls were used for gating to subtract autofluorescence and non-specific binding. Note that samples stained with DBA were only verified using negative controls, this is because it is a lectin and therefore has no isotype control. APC-Flag-positive cells were further sorted for either PE-CD31, FITC-F4/80 or 488-DAP.

For statistical analysis, we used GraphPAD Prism version 7 (GraphPad Software). Mean and SD were calculated based on standard settings in the case of normally distributed values, to estimate the average expression of FACS-detected signals after autofluorescence exclusion.

## 3. Results

### 3.1. Math6 Protein Can Be Detected in Murine Placenta

From the described phenotype of murine Math6 mutants, Math6 protein is predicted to be expressed in the placenta [15]. *Math6* mRNA has previously been identified in placental tissues. In contrast, all attempts to detect Math6 protein have so far remained unsuccessful. To close this gap, the present study aims to establish an immunohistochemical protocol that enables precise localization of Math6 protein and facilitate the detection of cell-type-specific Math6 expression within the placenta.

To localize the protein, we used mice in which Math6 was tagged with a Flag-sequence, performing an optimized protocol for immunohistochemical staining to locate this epitope successfully for the first time.

For analysis of the Math6 expression pattern, placentas were collected at critical stages of pregnancy, E10.5 and E13.5, and sections were subjected to immunohistochemical staining. The best results were achieved when tissues were fixed in 45 °C prewarmed 4% PFA in PBS. In contrast to our newly established protocol that provides specific and distinct staining, tests with other fixatives and staining with anti-Math6 or anti-Flag antibodies did not yield any results on paraffin sections of mouse tissues. All sections shown were stained with primary anti-Flag antibody from mice (F1804, Sigma-Aldrich). No primary antibody controls were performed on tissues from Math6 Flag-mice and wildtype mice. No unspecific staining occurred. Tissues from the Flag-mice revealed a bright positive signal in the cytoplasm of the cells in maternal and fetal tissues of the placenta.

**Figure 1:**
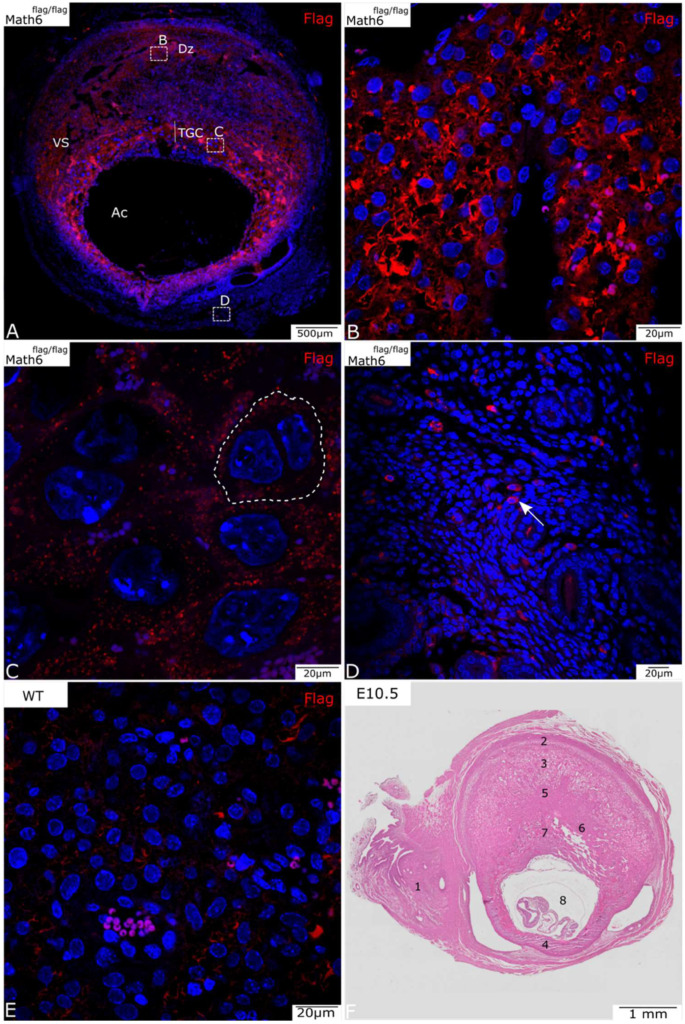
Math6-Flag expression in the murine placenta at E10.5 (**A**–**D**). (**A**) A complete section of a placenta shows all placental layers. Broken-lined boxes with letters indicate the areas from which details B, C, and D were taken. (**B**) Math6 is highly expressed in the decidua basalis (Dz) region below the negative myometrium. (**C**) Trophoblast giant cells (TGCs) also express Math6 (membrane visualized by dotted line). (**D**) Single positive cells can be found in the mesometrium (arrow). (**E**) The placenta of a wildtype mouse shows no specific staining but indicates non-specific background staining. (**F**) HE-stained placenta on E10.5 that serves as an overview. (**1**) Mesometrium, (**2**) MLAp, (**3**) mesometrial decidua (basalis), (**4**) anti-mesometrial decidua, (**5**) junctional zone, (**6**) labyrinth, (**7**) TGC, (**8**) amniotic cavity (Ac) with embryo, vascular sinuses (Vs).

Placentas from wildtype mice lacking the Flag-tag were also subjected to immunohistochemical staining against the Flag-epitope as a negative control. In the wildtype placenta, a negligible non-specific signal was detected, which could be further reduced by our method optimization (Figure 1E). 

### 3.2. Math6 Is Expressed in Various Regions of the Murine Placenta

**Figure 2:**
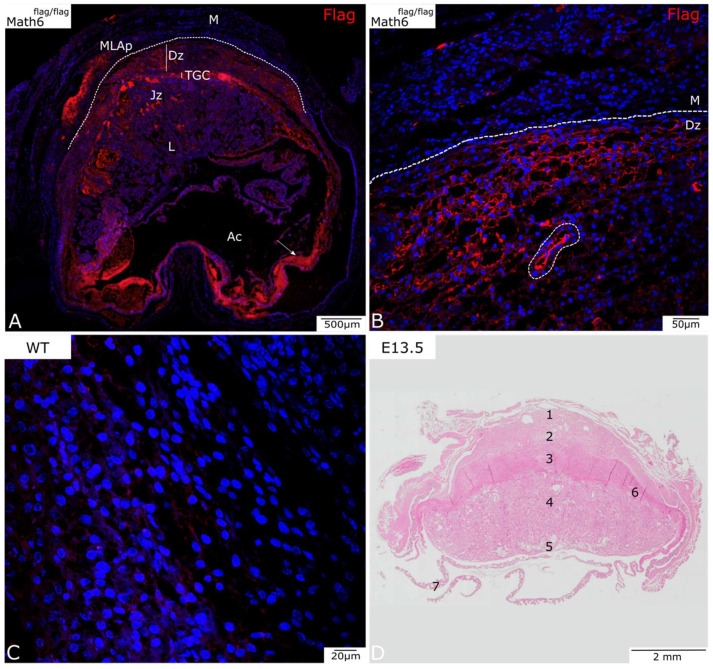
Analysis of Math6 expression in Math6-Flag mice on day E13.5 (**A**,**B**). (**A**) A complete section of placental on day E13.5 with the amniotic cavity (Ac) visible. (**B**) The myometrium (M) and the decidua basalis (Dz) at a higher magnification. A blood vessel can be observed in this area (encircled with dotted line). (**C**) Wildtype control (**D**) HE-stained placenta on E13.5 that serves as an overview. (**1**) MLAp, (**2**) mesometrial decidua (basalis), (**3**) junctional zone, (**4**) labyrinth, (**5**) umbilical cord, (**6**) TGC, (**7**) yolk sack. Trophoblast giant cells (TGCs), junctional zone (Jz), labyrinth (L), mesometrial lymphoid aggregate of pregnancy (MLAp).

At E10.5 and E13.5 of placental development, a positive antibody signal was detected in several areas of the placenta, in the maternal and fetal components (Figure 1 and Figure 2).

Figure 1A shows an overview of the placenta on development day E10.5. Besides the immunohistochemical staining for Flag (Figure 1, red), the nuclei were stained with DAPI (blue) for better visualization of individual cells. At this stage of development, Math6 appears to be expressed primarily in the decidua (Figure 1B), TGC (Figure 1C) and cells of the mesometrium (Figure 1D).

The placenta shows a strong signal in the decidua basalis, in particular around the vascular sinuses and near the MLAp, which is localized in the uterine wall (Figure 1A). The protein seems to be predominantly expressed in the peripheral region of the cytoplasm in many, but not all, cells in this section of the placenta. Even though the signal is quite bright, it is not so well defined, thus making it difficult to identify single positive cells. Myometrial smooth muscle cells surrounding the decidua do not express Math6.

The fetal layers of the placenta, which include the TGC and the spongiotrophoblast layer, both show detectable Math6 levels. TGCs, which can be recognized by their characteristic larger size and characteristic location between the decidua and the labyrinth, also show Math6 expression (Figure 1C). The fluorescence signal can be detected throughout the whole cytoplasm of these cells. Compared to decidua cells, the individual TGC are more distinguishable because their cell borders are partially visible (Figure 1C, dotted line).

Math6-positive single cells can also be found in the mesometrium (Figure 1D), which mostly consists of connective tissue and smooth muscle cells for stabilization of the uterus. These cells can primarily be found in blood vessels (Figure 1D, arrow).

Figure 2A shows an overview of the placenta on development day E13.5. The Flag protein is similarly visualized in red, and the nuclei were counterstained in blue using DAPI.

At E13.5, the placenta further expresses Math6 in TGCs and the decidua basalis, as observed at E10.5. At this stage of development, where the junctional zone is further expanded, Math6 expression is also observed in this region. The labyrinth, on the other hand, seems to be mostly negative for Math6 expression. 

The MLAp is also further developed and shows high expression of Math6 in the marginal area.

Figure 2B shows the myometrium and decidua basalis at a higher magnification. This shows the passage from Math6-negative myometrium and below-lying positive decidua basalis. Furthermore, a blood vessel is observed in this area, indicating possible Math6-positive endothelial cells.

Structures observed in the amniotic cavity include the yolk sack and collapsed myometrium, as the embryo was removed for faster fixation of the tissue. The detected signal in this region (Figure 2A, arrow) appears due to autofluorescent erythrocytes at a higher magnification. As this is considered a non-specific signal, it will not be discussed further.

### 3.3. More than 5 Percent of the Murine Placental Cells Express Math6

**Figure 3:**
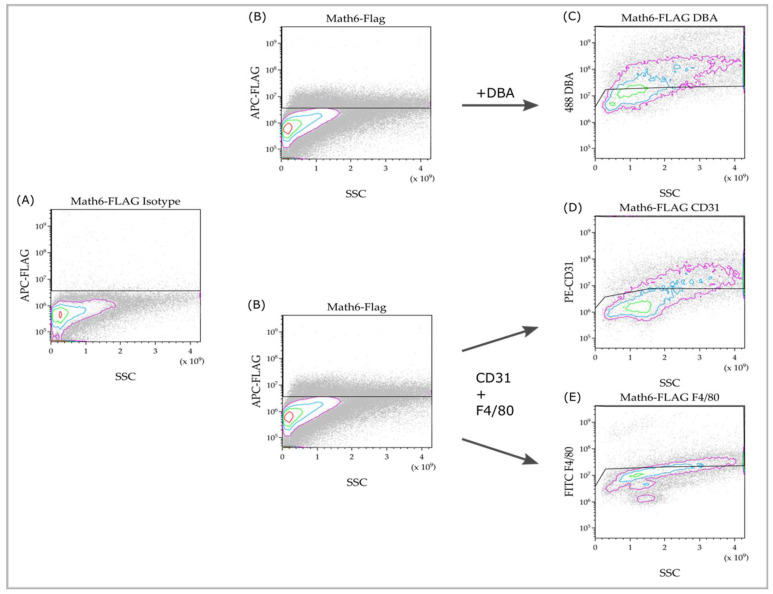
Flow cytometry of Math6-stained cell suspensions (**B**) compared to the isotype control (**A**), and the subpopulation analysis within the Math6+ subset (**C**–**E**). The cell population is represented by individual cells depicted as dots. The highest cell density is indicated by the innermost red circle, with the density decreasing towards the outer pink circle. All cells passing the gating line are considered positive for the respective investigated marker.

To characterize the Math6-expressing cell population(s) within the murine placenta, flow cytometry was used to investigate a single-cell suspension made from placental tissue at the developmental stage E10.5. From each animal, one sample remained unstained, while another was stained with isotype-control antibodies to determine and optimize the gating parameters (line in Figure 3A).

Math6 expression was detected via staining against the Flag-epitope (Figure 3B). In contrast to the negative control (Figure 3A), a specific positive signal can be detected. The Math6+ cells do not appear to belong to a homogeneous group of cells, as the stained cells vary in size and granularity. As a result of this, it can be concluded that Math6 is expressed in multiple distinct cell populations.

We were further interested in cells that can be detected via CD31 (endothelial cells), F4/80 (macrophages) or DBA (uNK). These cell types were of particular interest as they are significantly involved in the vascularization process of the placenta, which is deficient in knockout mice. Since they can be identified via double staining, rather than by size or localization like TGCs, they were studied using FACS.

The markers, CD31, F4/80 and Flag, were detected through triple staining, whereas double staining was performed for DBA and Flag. These subpopulations analyses can be seen in Figure 3C–E. In comparison to the negative controls, specific signals were detected here. This means Math6+CD31+, Math6+F4/80+ and Math6+DBA+ cells are present in the placenta and can be detected through flow cytometry.

**Figure 4:**
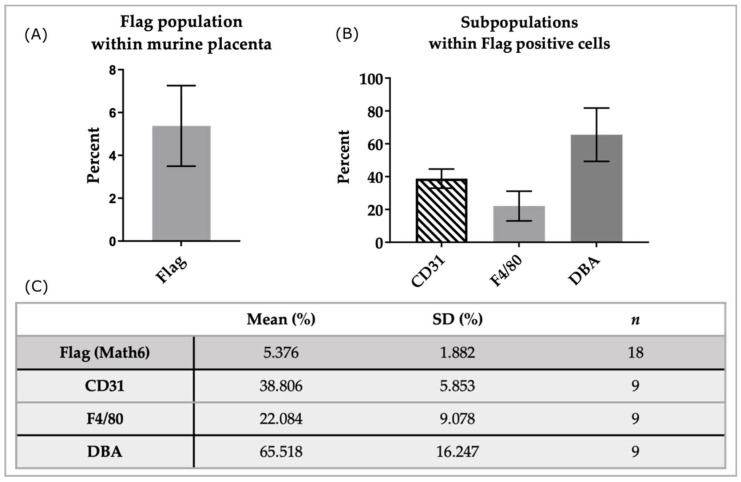
Analysis of subpopulations in Math6-positive cells of the murine placenta. Shown here is the population of Flag+ (Math6) cells from all cells analyzed (**A**) as well as the subpopulations within the Flag+ cells (**B**). The table (**C**) presents the analysis results as the mean, standard deviation (SD), and the number of samples analyzed (*n*).

In the analyzed samples (*n* = 18), an average of 5.38 ± 1.89 percent of cells were positive for the Flag-tag, thus detecting Math6 (Figure 4A). A subset of Math6-positive cells was further analyzed through triple staining with anti-CD31 and anti-F4/80 (*n* = 9). Within the population that expressed Math6 an average of 38.81 ± 5.84 percent also expressed CD31. Another subpopulation of 22.08 ± 9.08 percent in the group of Math6-positive cells was also positive for F4/80 (Figure 4B,C).

Furthermore, Flag-positive cells, which express Math6, were double-stained with DBA lectin (*n* = 9). Flow cytometric analysis showed that 65.52 ± 16.23 percent of Flag-positive cells were positive for α-linked N-acetylgalactosamine, detected through DBA.

When examining the experimental results obtained from the placentas of only one animal at a time, the standard deviation was significantly reduced. For example, in the DBA+ group, the SD decreased from 16.25 to 2.78 (Table 2).

## 4. Discussion

Math6 expression is essential for placental development, as maternal Math6 knockout is associated with impaired decidualization and vascularization, leading to pregnancy loss [15]. This study provides the first insights into the main cell types expressing Math6 protein in the murine placenta using transgenic Math6 Flag mice. Thus, we were able to localize Math6 expression via immunohistochemistry, and we detected CD31+Math6+, F4/80+Math6+, and DBA+Math6+ cells by fluorescence-activated cell sorting (FACS) analysis in the murine placenta. By combining our results with the knowledge about the Math6 knockout phenotype described previously, we gained significant progress towards elucidating the underlying cause. The severe failures in placental development in mice that lack Math6 appear to be caused by complex crosstalk between multiple cell types. This crosstalk among the cells identified as Math6+ needs to be further investigated in the future to provide definitive insights into the pathomechanisms in Math6-knockout mice.

For the first time, transgenic mice were used for the detection of Math6 through immunohistochemistry in placental tissues. Divvela et al., who established these mice, have already proven that specific signal detection for Math6 is possible in the cells of these mice [16]. We were able to reproduce these results in the placental tissues of Math6 Flag-tag mouse. Placental tissue is a difficult organ for immunohistochemistry [3]; however, we established an improved protocol for immunohistochemistry that shows specific and distinct staining. From this, we provided the possibility for Math6 expression analysis, giving the opportunity to carry out double staining. Additionally, our protocol reduces background staining as much as possible, whereby almost no staining is visible in the Flag-tag-negative wildtype mice used as the negative control.

We discovered that the expression of Math6 protein on days E10.5 and E13.5 can be found in both the maternal MLAp and decidua as well as in the fetal TGCs, labyrinth and junctional zone of the placenta. Within all these areas and cell types, Math6 expression is primarily seen in the cytoplasm of the cells. These results were expected given that we previously demonstrated that Math6 is localized to the cytoplasm of several early embryonic tissues, including the inner cell mass of blastocysts [17] and in fibroblasts during reprogramming [16].

The Math6 protein distribution in the decidua basalis is striking because in most areas of the decidua, all cells seem to express the protein. A high expression in the maternal portion of the placenta was expected, as the phenotype is influenced by the maternal genotype in the Math6-knockout mouse, thus suggesting a defect in maternal cells. In line with our new findings, our previous results showed *Math6* expression in all layers of the tissue through in situ hybridization [15]. Those prior analyses of location suggest that decidua cells are positive for *Math6*, which was also proven by Ma et al., who showed that *Math6* is upregulated in polyploid decidua cells [18]. We were able to demonstrate that this previously shown gene expression domain correlates with the protein distribution of Math6. Further research should be undertaken to investigate whether Math6 RNA and protein expression correlate in other organs, especially if they do not show phenotypic abnormalities in knockouts.

By immunohistochemical staining, we were also able to detect TGCs as another Math6+ cell type. In general, the group of bHLH transcription factors has a key role in the development of TGCs. One factor being very important for promoting TGC differentiation is Hand1 [19]. In contrast, other bHLH factors like Mash2 have antagonizing effects, and Mash2 mutants show an increased TGC number [19,20]. Even though there is evidence of Math6 expression promoting cell proliferation [21,22], TGC number is increased in Math6-knockout mice, thus showing a deficient fetal–maternal interface [15]. However, as we have shown, Math6 is expressed in diverse cell types, which may interfere with the crosstalk between cells that stimulate TGC proliferation.

For expanding the investigation possibilities regarding the involvement of Math6 in the murine placental developmental processes, we established a protocol for FACS analysis. Cell suspensions were isolated from fresh placental tissue from day E10.5, and we identified that more than 5% of the FACS-sorted cells were Math6+. The parameters within one animal were more homogeneous, not only for Flag-positive cells but for every detected cell type (Table 2). This is caused by the fact that, in addition to the normal variations seen in biological replicates, mating can only be restricted to a specific time window, and the course of pregnancy adds additional fluctuations. During interpretation of the results, it must be additionally considered that only cells smaller than 40 µm were analyzed in our experiments. Information about larger cells, such as TGCs, cannot be obtained by FACS. It can thus be estimated that more than 5% of all cells of the placenta express Math6. This also leads us to the fact that it was not possible to show Math6+ TGCs with FACS. Furthermore, other big cells, like decidua cells, may be less prominent in the collected cell suspension than in the placenta, thus leading to a reduced count of Math6+ cells. Therefore, we assume that the actual number of Math6+ cells is likely to be higher than what was measured by FACS. Nevertheless, the detected Math6+ cells could be further analyzed through staining with other markers in this project or sorted for further downstream applications in the future.

Uterine natural killer cells, that we detected with DBA lectin [9], formed a subpopulation of 65.52% within the 5% FACS-sorted Math6+ cells, labeled as Math6+DBA+ cells. This cell type, providing up to 70% of the leukocytes during mid-gestation [23], plays a key role in vascular remodeling and decidua development [24], and it is less expressed in Math6-knockout mice [15]. Since uNK-deficient mice do not show the same devastating effects on fertility [25], interaction with other Math6+ cells altered in knockout mice seems to be the cause of the problem. It is also questionable whether the loss of Math6 alone limits uNK proliferation or whether this is related to the endocrine crosstalk with other stimulating cells such as decidua cells. Nevertheless, our results fit with the existing literature that describes possible Math6+ uNK cells detected based on location through in situ hybridization [15].

Macrophages, which we detected through F4/80 [10], formed a subpopulation of 22.08% within the FACS-sorted Math6+ cells. Our attention was drawn to this cell type as they are the second predominant leukocyte type in the placenta [26]. Right now, there are no data relating to Math6 in the context of the immune system in the placenta; however, it is particularly important not only for fetus tolerance but also for correct placental development. Macrophages secrete growth factors and cytokines, such as interleukin 10 (IL10) and endothelial growth factor (VEGF), and help establish the maternal–fetal interface, trophoblast invasion, and angiogenesis [27,28]. All of these mentioned processes are disturbed when Math6 is missing [15], and this could be due, among other circumstances, to the macrophages. With the help of flow cytometry, these cell types could be studied in more detail; for example, to see if their numbers are altered in Math6-knockout mice, like uNK or TGCs. In further studies in immunodeficient mice, it would also be interesting to identify possible interactions with Math6.

The third subpopulation we detected was CD31+ cells that made up 38.8% of the FACS-sorted Math6+ population. CD31, an important marker for vascularization, is predominantly expressed on endothelial cells. As we observed cells with an endothelial phenotype when immunostaining against Math6, proved this assumption and showed Math6+CD31+ cells. In accordance with our investigation, a study published in 2014 revealed that Math6 is indeed not only expressed but upregulated during endothelial determination [14]. It was also already proven that Math6 is expressed in human endothelial cells and acts as a sheer-stress-reactive bHLH factor [14]. The bHLH domain of Math6 also induces degradation of HIF-1α and HIF-2α, resulting in HIF-2α-selective target genes being antagonized in the cell culture of ECs [29]. Therefore, it remains questionable whether Math6–HIF interaction is involved in the mouse placenta or whether it can be compensated by other factors. Our results also fit with published data of Math6-knockout mice, which show that *CD31* is significantly reduced [15]. Since vascularization and angiogenesis is highly defective in these mutant mice, this also suggests endothelial cell involvement.

Since surface markers were partly stained separately due to spectral overlap, the percentage results must be interpreted individually. It must also be noted that DBA lectin is not specifically manufactured for FACS application. Moreover, the gating panel can only be adjusted with negative controls, which could allow for increased autofluorescence, thus resulting in an increased number of possible DBA+ cells. Nevertheless, complete autofluorescence can be excluded via a comparison with negative controls and the large number of measured DBA+ cells. In addition, Math6+DBA+ cells were also detected by immunostaining.

All in all, FACS analysis opens up the possibility of analyzing sorted Math6+ cells. These cells can also be sorted and used for further downstream applications if indented.

## 5. Conclusions

To conclude, our study provided two newly established protocols that help circumvent the lack of a highly specific antibody against Math6. Using these methods, we were able to reproduce and confirm some of the previous findings about *Math6* expression. Furthermore, we have been able to confirm that the location of *Math6* RNA, shown by Böing et al. [15], and protein expression correlate with each other. In contrast to previous results based on analysis of cell locations, we evaluated double staining against cell-specific markers to detect double expression with Math6, identifying Math6+DBA+ cells. Furthermore, new cell types expressing Math6 in the placenta, such as TGCs, macrophages (Math6+F4/80+) and endothelial cells (Math6+CD31+), were identified through this work. This may help us to examine the possible disturbed crosstalk between cells typically expressing Math6 in knockout mice. For now, these results have yielded more detailed information about a protein that leads to reproductive-insufficient females when missing and, therefore, the molecular pathomechanisms of spontaneous abortions in mice.

## Figures and Tables

**Table 1:** Staining single-cell suspensions isolated from fresh placental tissues. Isotype and negative controls were fixed and permeabilized similarly for more accurate gating conditions. DBA was stained separate from CD31 and F4/80 due to extraction spectra overlap. Allophycocyanin (APC), phycoerythrin (PE), fluoresceinisothiocyanat (FITC).

	(1) Antibody for Extracellular Antigen	(2)	(3)	(4) Antibody for Intracellular Antigen
Negative control	-	Fixation (15 min, RT)	Permeabilization (10 min, RT)	-
Isotype control	APC Rat IgG2a, λ and PE Rat IgG2b, κ and FITC Rat IgG2a, κ			-
Flag	-			Rat-Anti DYKDDDDK (Flag) conjugated with APC
Flag + DBA	DBA lectin			Rat-Anti DYKDDDDK (Flag) conjugated with APC
Flag + CD31 + F4/80	Rat Anti-CD31 conjugated with PE and Rat Anti-F4/80 conjugated with FITC			Rat-Anti DYKDDDDK (Flag) conjugated with APC

**Table 2:** FACS analysis results in percentages.

Mouse	Flag Cells in %	CD31 Cells in % within Flag Population	F4/80 Cells in % within Flag Population	DBA Cells in % within Flag Population
F736	5.26	36.96	27.15	
	6.85	35.5	26.84	
	5.25	41.58	34.31	
	6.71	41.4	29.39	
	8.28			52.06
	7.23			64.28
	7.08			64.44
F783	2.48	31.24	19.23	
	2.86			39.44
	2.93			47.91
F701	5.92	34.93	11.23	
	6.02	35.84	9.98	
	5.06	40.26	12.27	
	6.73			82.81
	6.77			84.9
	5.81			78.25
F711	3.01	51.54	28.36	
	2.16			75.57

## Data Availability

Data sharing not applicable.
